# Supplementary material for: An Efficient Method for Selective Syntheses of Sodium Selenide and Dialkyl Selenides
Source: Molecules. 2022 Aug 16;27(16):5224. doi: 10.3390/molecules27165224 (PMC9414418; doi:10.3390/molecules27165224)
Supplement: Supplementary file 1 [file molecules-27-05224-s001.zip › molecules-1851933-supplementary.pdf]

Supplementary Materials

for

An Efficient Method for Selective Syntheses of Sodium Selenide and Dialkyl Selenides

Na Hye Shin †, Yoo Jin Lim †, Chorong Kim, Ye Eun Kim, Yu Ra Jeong, Hyunsung Cho, Myung-Sook Park, and Sang Hyup Lee *

College of Pharmacy and Innovative Drug Center, Duksung Women's University,

Seoul 01369, Korea

*sanghyup@duksung.ac.kr

^{1}H and ^{13}C NMR spectrum of compound **1a** S2

^{1}H and ^{13}C NMR spectrum of compound **1b** S3

^{1}H and ^{13}C NMR spectrum of compound **1c** S4

^{1}H and ^{13}C NMR spectrum of compound **1d** S5

^{1}H and ^{13}C NMR spectrum of compound **1e** S6

^{1}H and ^{13}C NMR spectrum of compound **1f** S7

^{1}H and ^{13}C NMR spectrum of compound **1g** S8

^{1}H and ^{13}C NMR spectrum of compound **1h** S9

^{1}H and ^{13}C NMR spectrum of compound **1i** S10

^{1}H and ^{13}C NMR spectrum of compound **1j** S11

^{1}H and ^{13}C NMR spectrum of compound **1k** S12
^{1}H and ^{13}C NMR spectrum of compound **1l** S13
^{1}H and ^{13}C NMR spectrum of compound **1m** S14

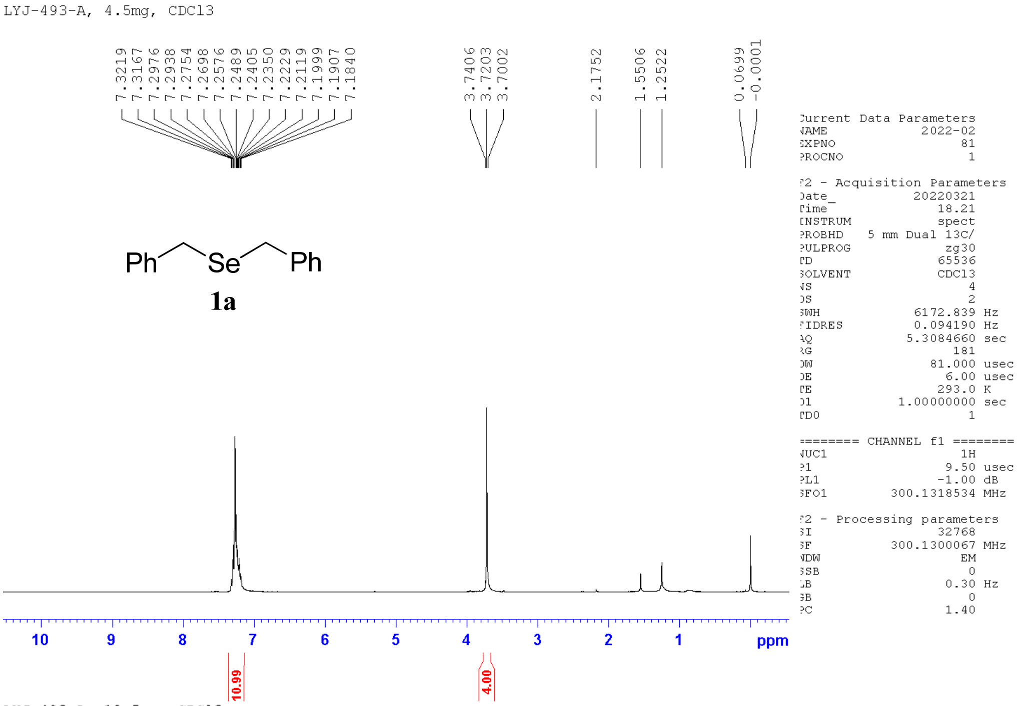

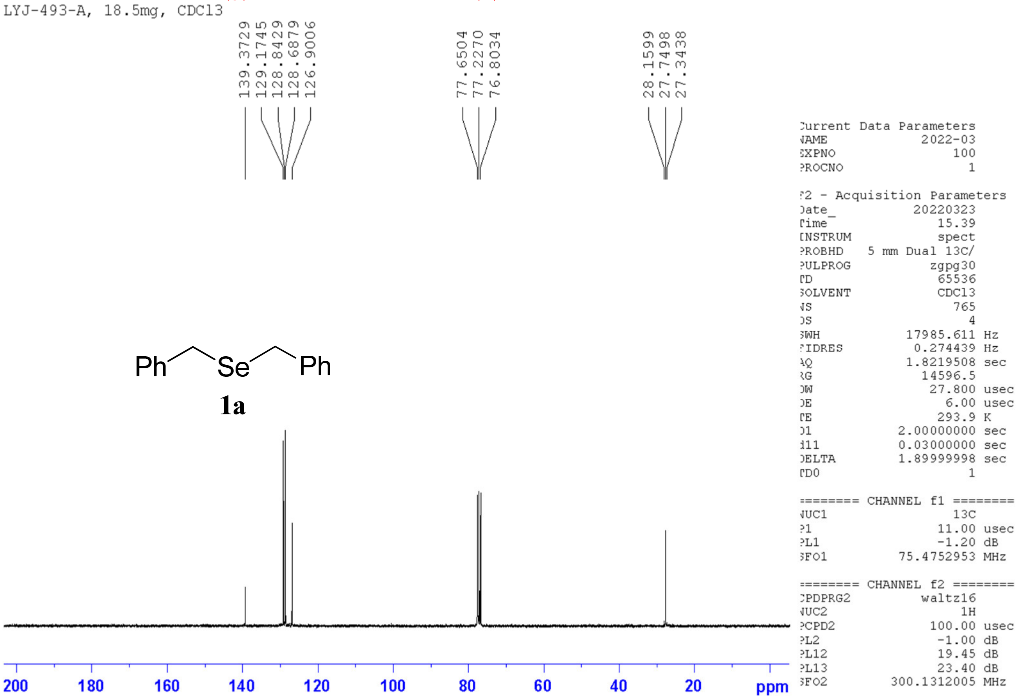

^{1}H NMR spectrum (300 MHz, $CDCl_3$) of compound 1a

^{13}C NMR spectrum (75 MHz, $CDCl_3$) of compound 1a

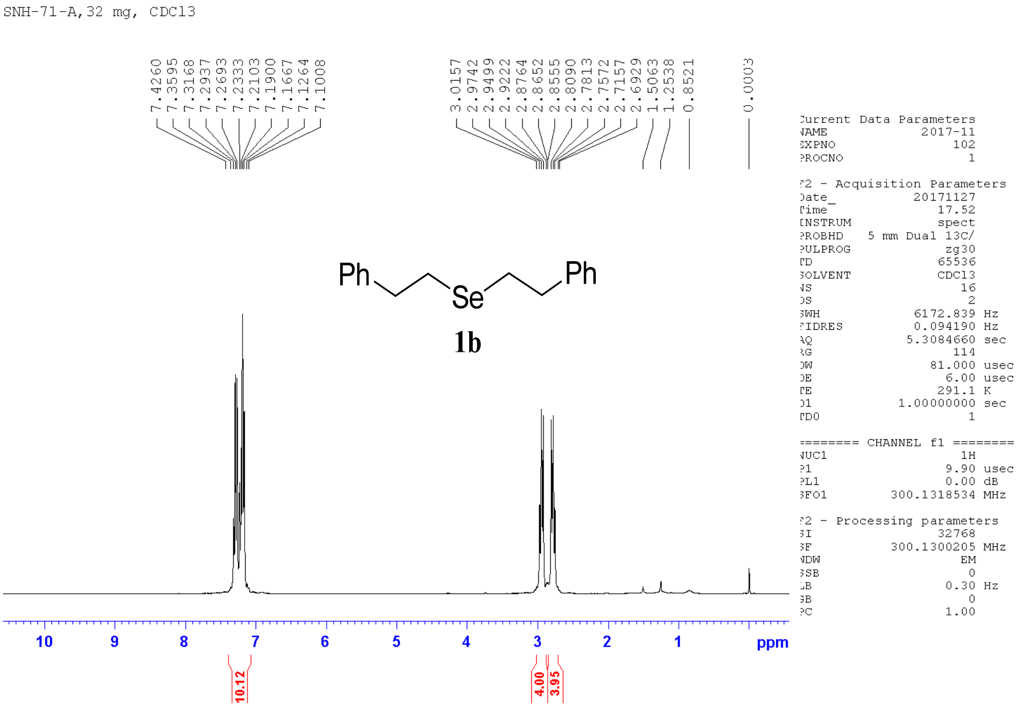

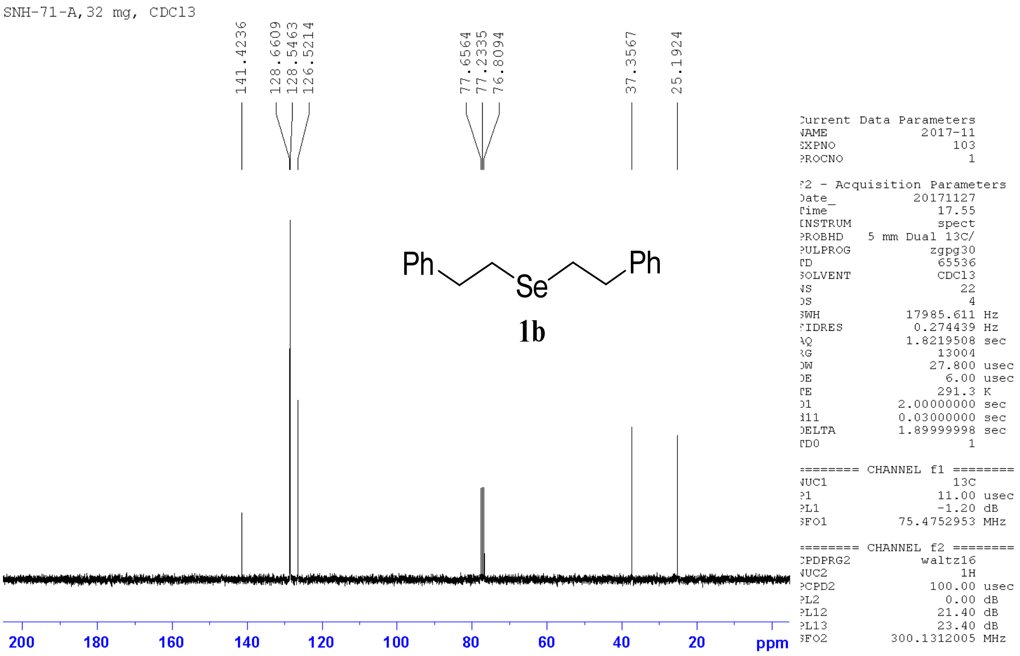

^{1}H NMR spectrum (300 MHz, $CDCl_3$) of compound 1b

^{13}C NMR spectrum (75 MHz, $CDCl_3$) of compound 1b

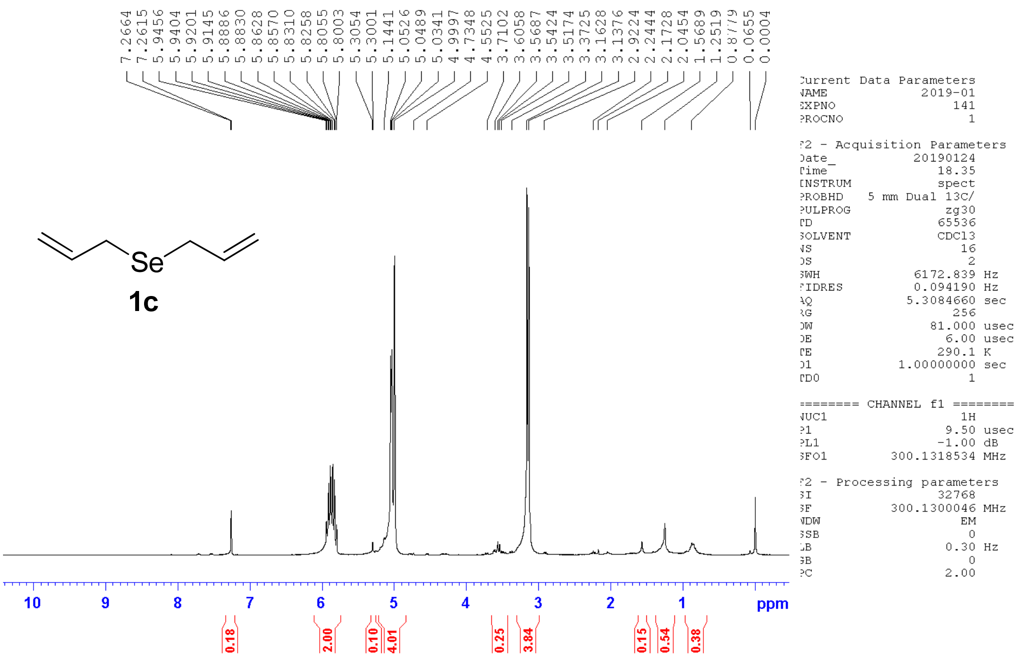

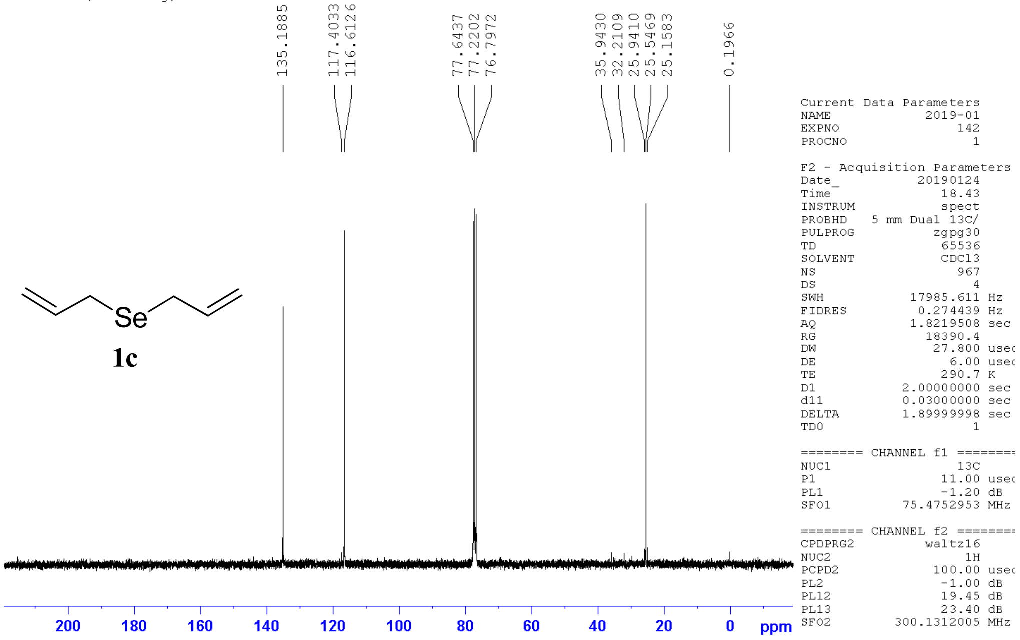

^{1}H NMR spectrum (300 MHz, $CDCl_3$) of compound 1c

^{13}C NMR spectrum (75 MHz, $CDCl_3$) of compound 1c

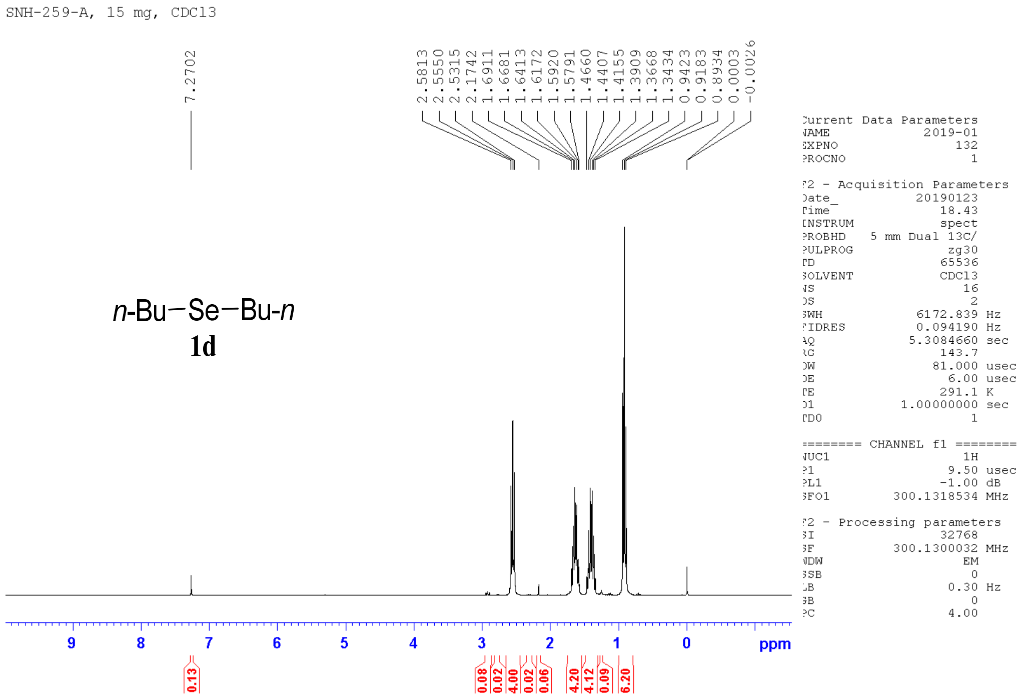

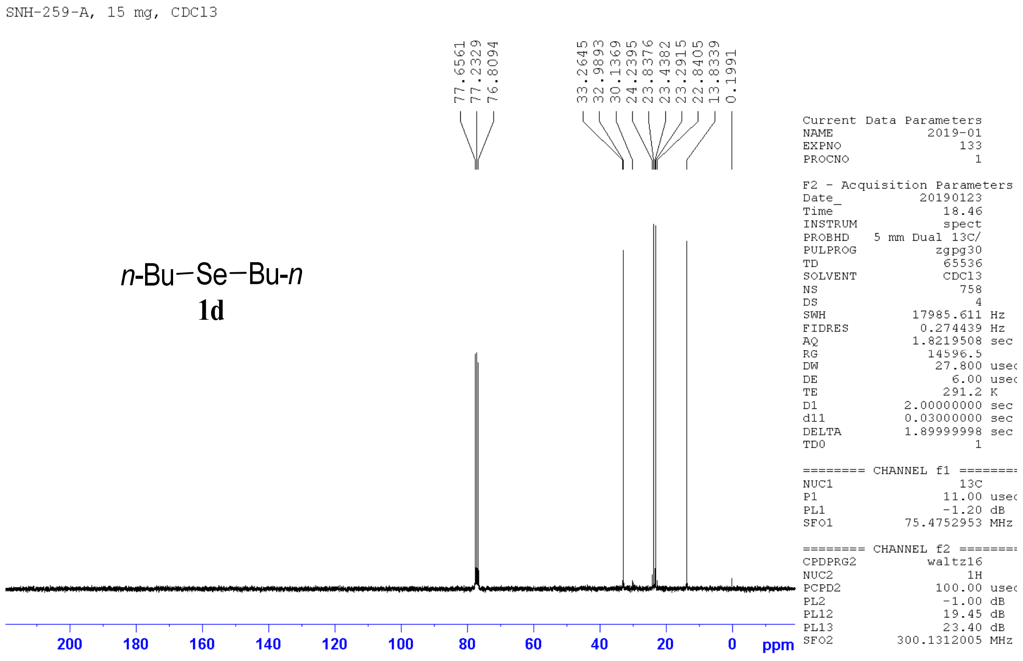

^{1}H NMR spectrum (300 MHz, $CDCl_3$) of compound 1d

^{13}C NMR spectrum (75 MHz, $CDCl_3$) of compound 1d

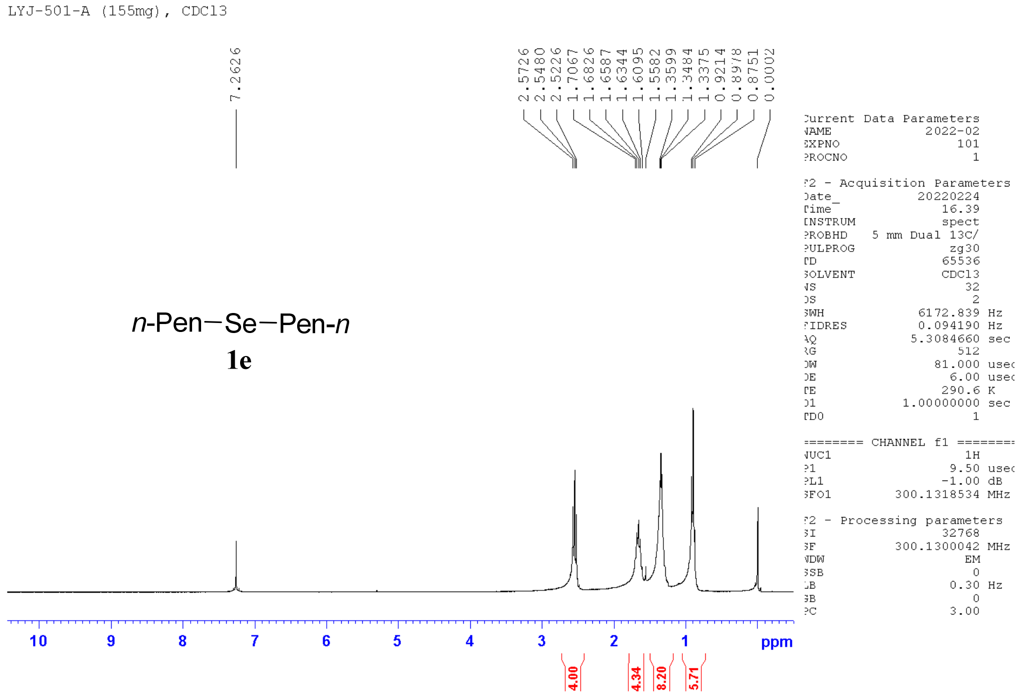

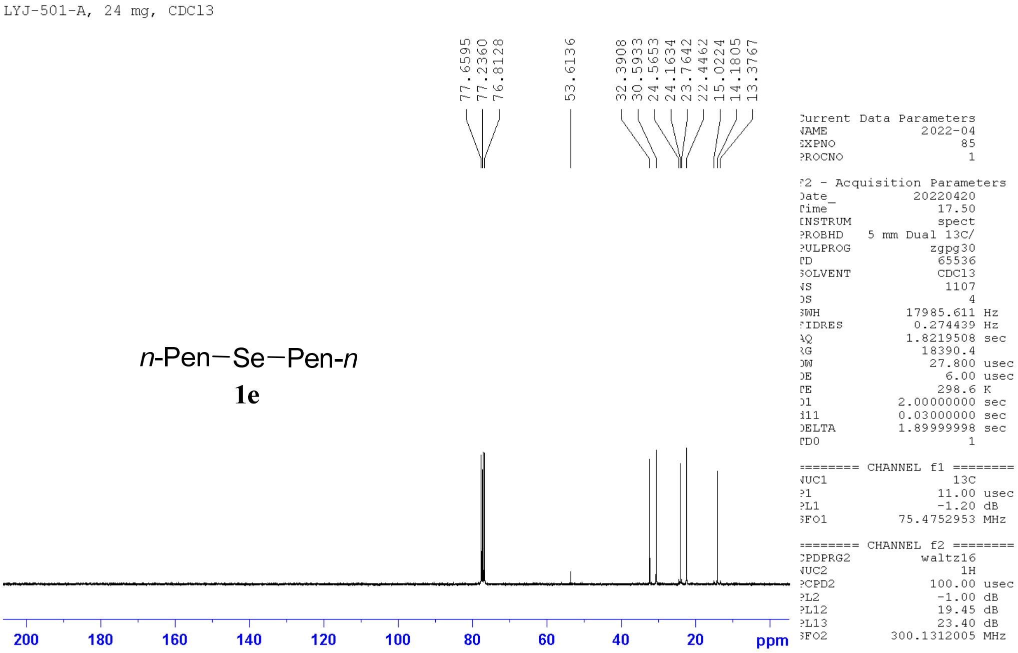

^{1}H NMR spectrum (300 MHz, $CDCl_3$) of compound **1e**

^{13}C NMR spectrum (75 MHz, $CDCl_3$) of compound **1e**

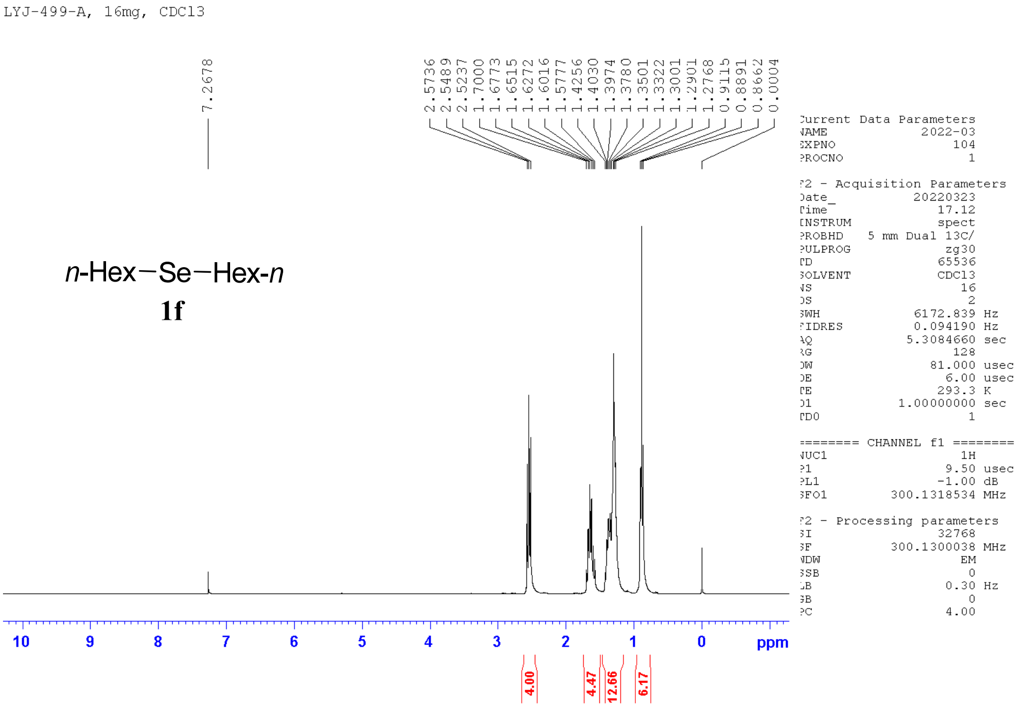

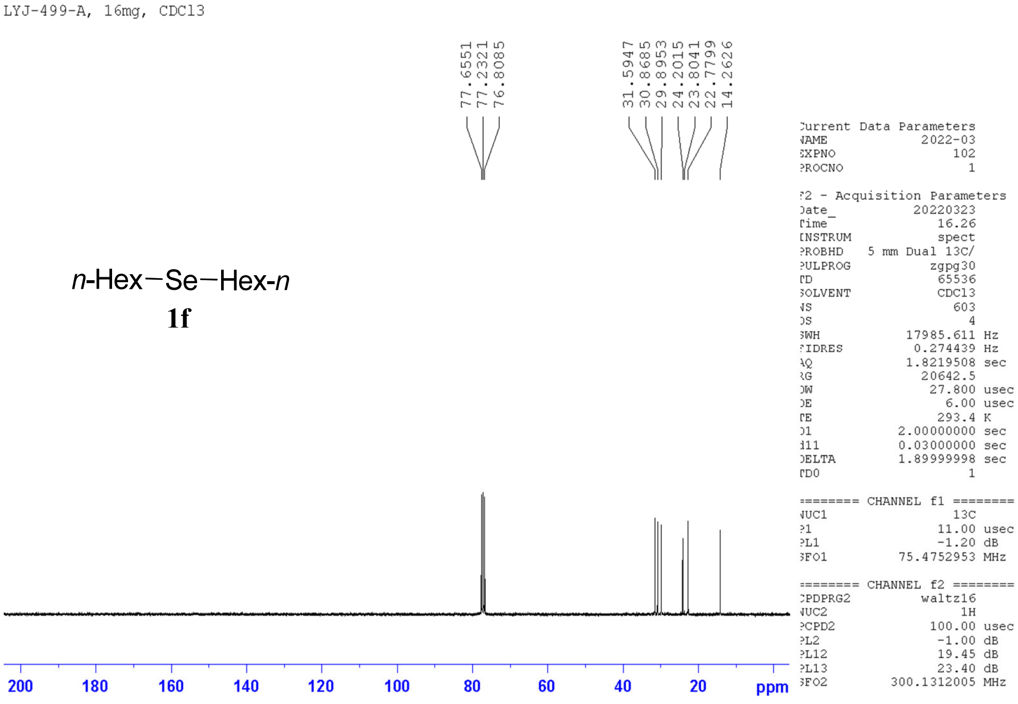

^{1}H NMR spectrum (300 MHz, $CDCl_3$) of compound 1f

^{13}C NMR spectrum (75 MHz, $CDCl_3$) of compound 1f

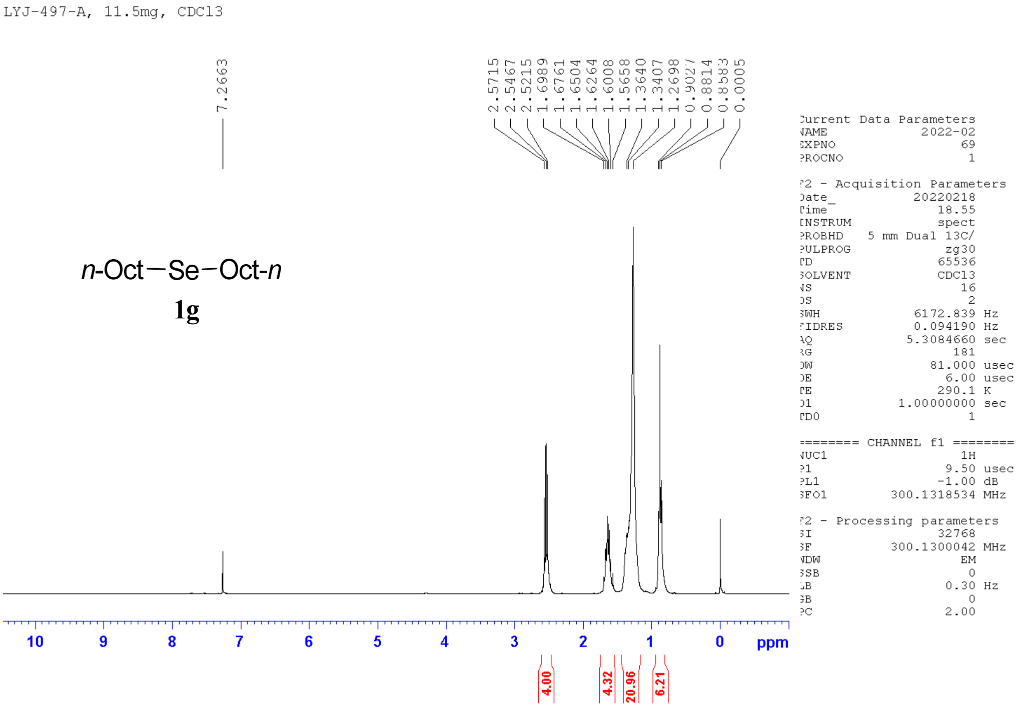

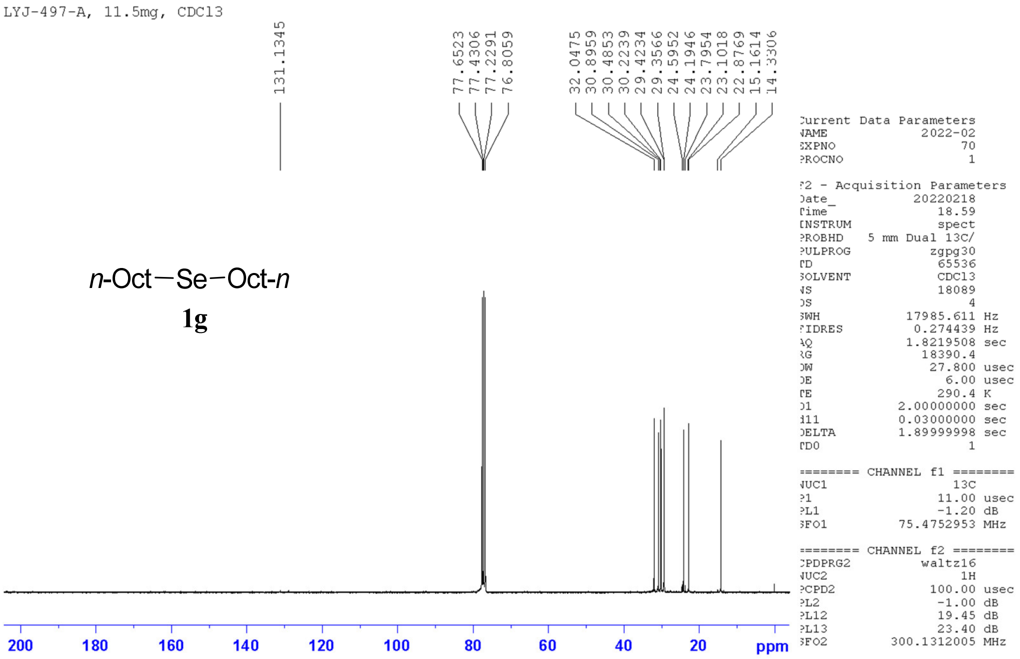

^{1}H NMR spectrum (300 MHz, $CDCl_3$) of compound 1g

^{13}C NMR spectrum (75 MHz, $CDCl_3$) of compound 1g

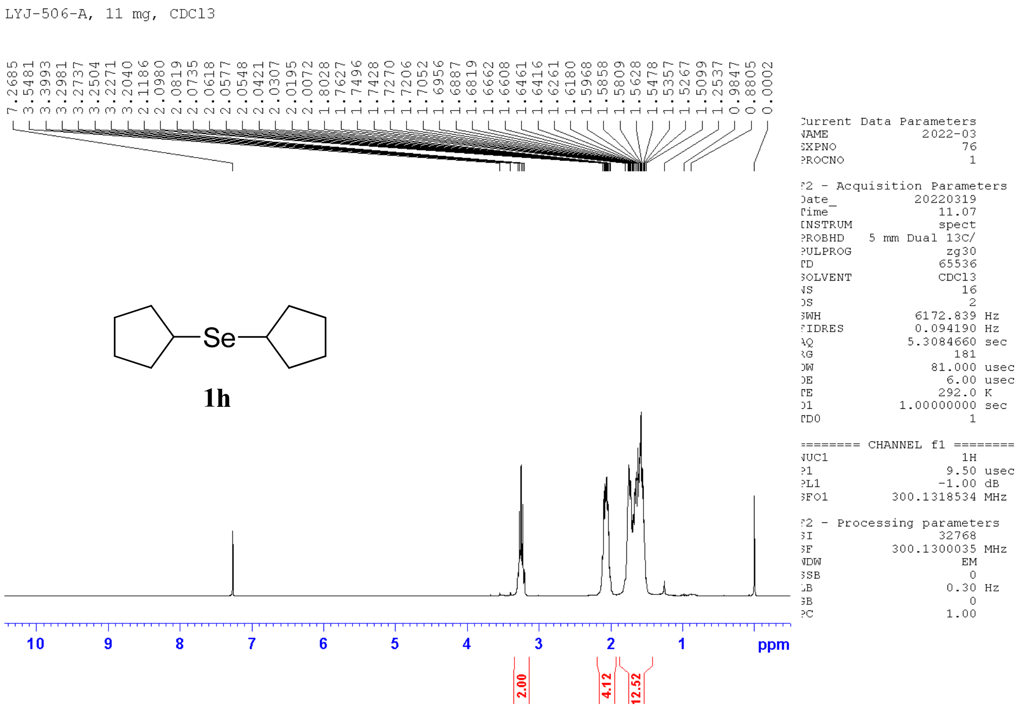

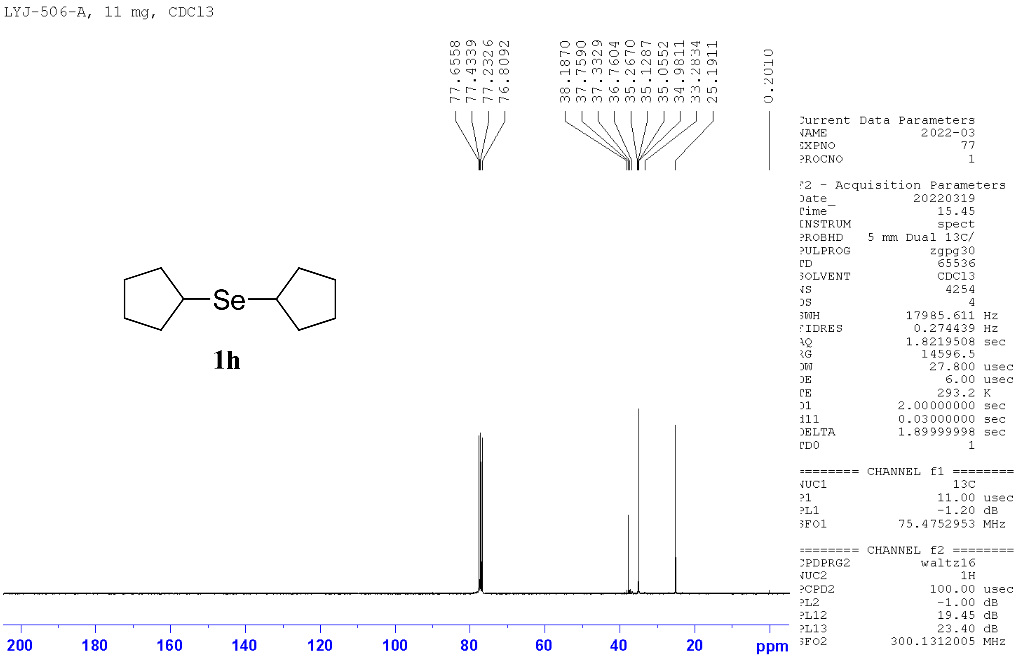

^{1}H NMR spectrum (300 MHz, $CDCl_3$) of compound 1h

^{13}C NMR spectrum (75 MHz, $CDCl_3$) of compound 1h

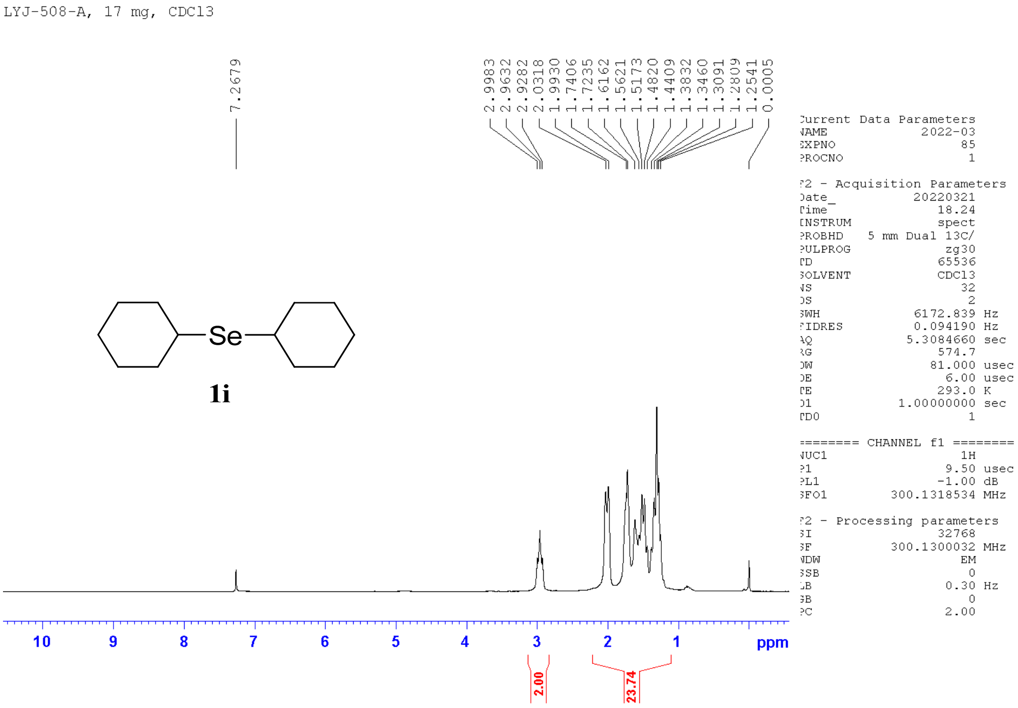

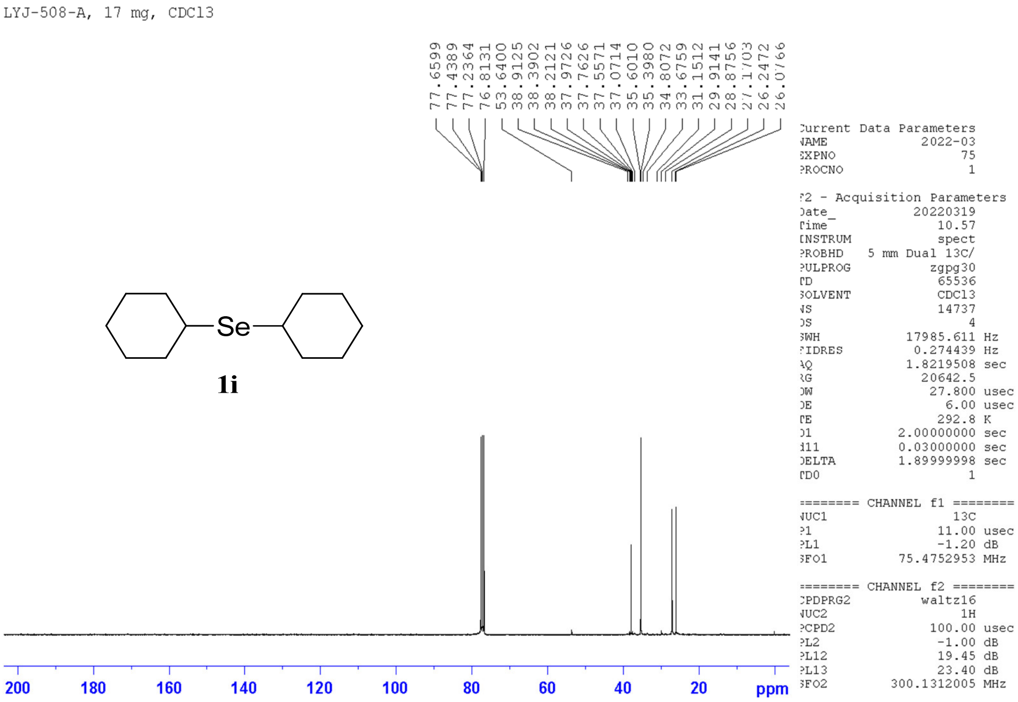

^{1}H NMR spectrum (300 MHz, $CDCl_3$) of compound 1i

^{13}C NMR spectrum (75 MHz, $CDCl_3$) of compound 1i

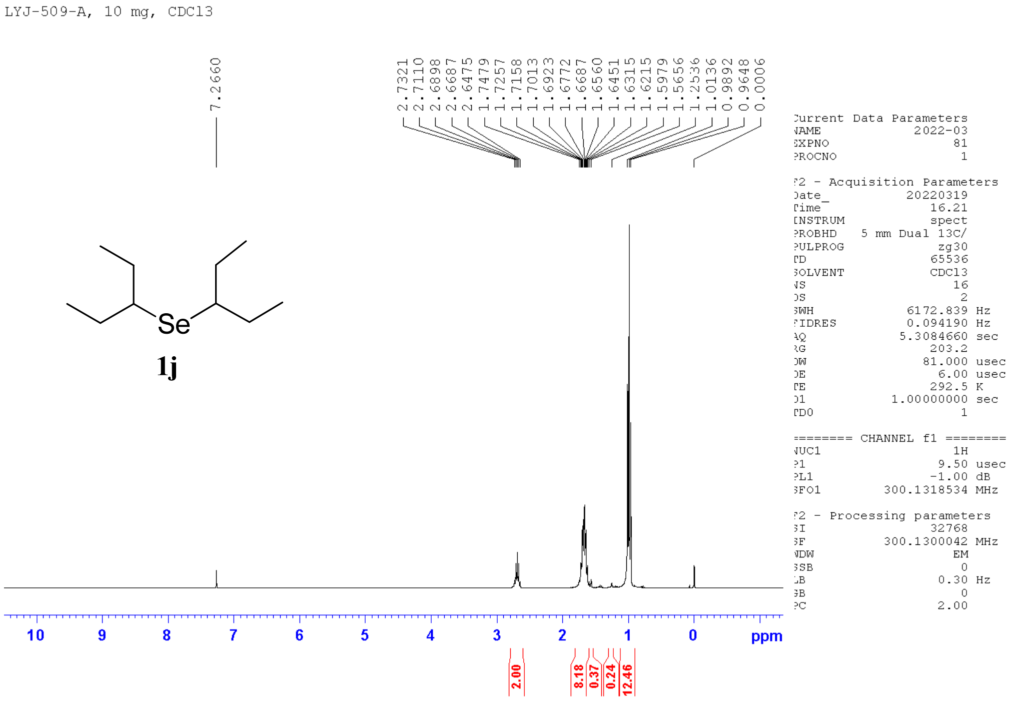

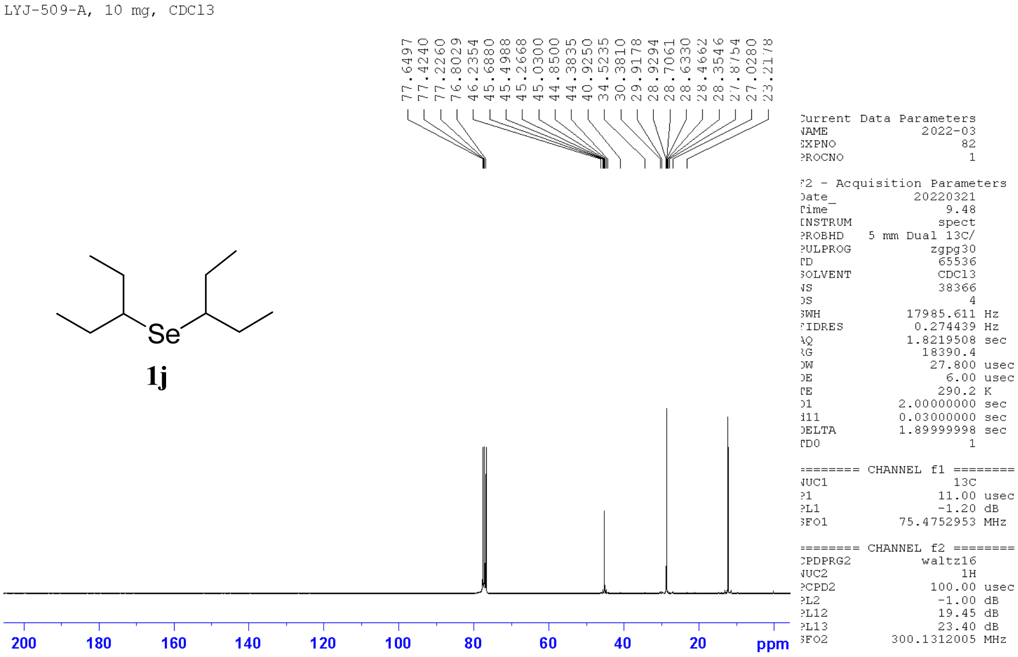

^{1}H NMR spectrum (300 MHz, $CDCl_3$) of compound 1j

^{13}C NMR spectrum (75 MHz, $CDCl_3$) of compound 1j

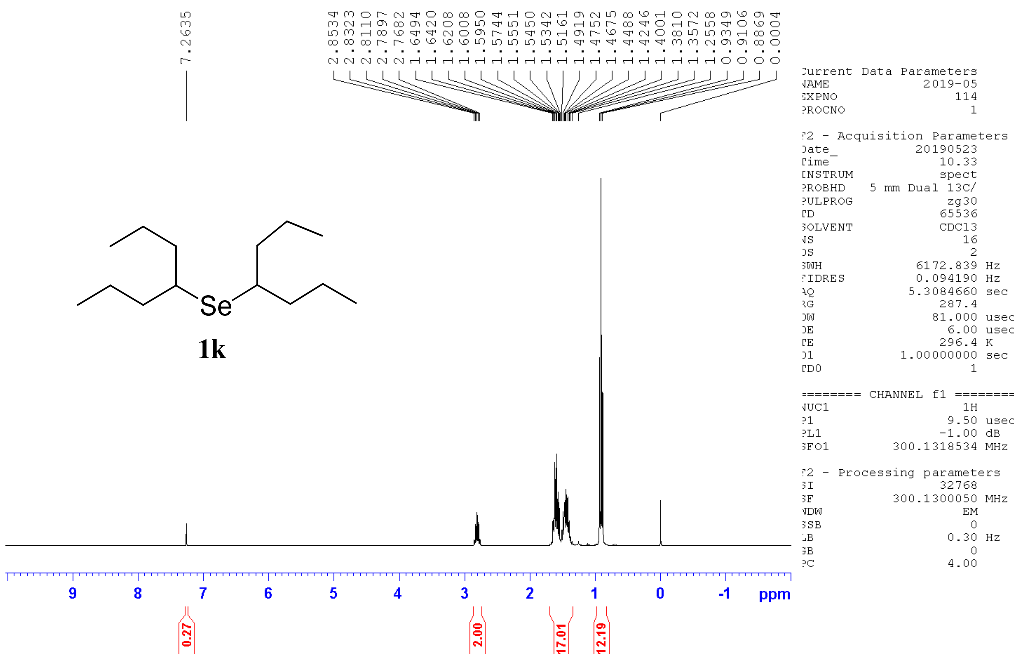

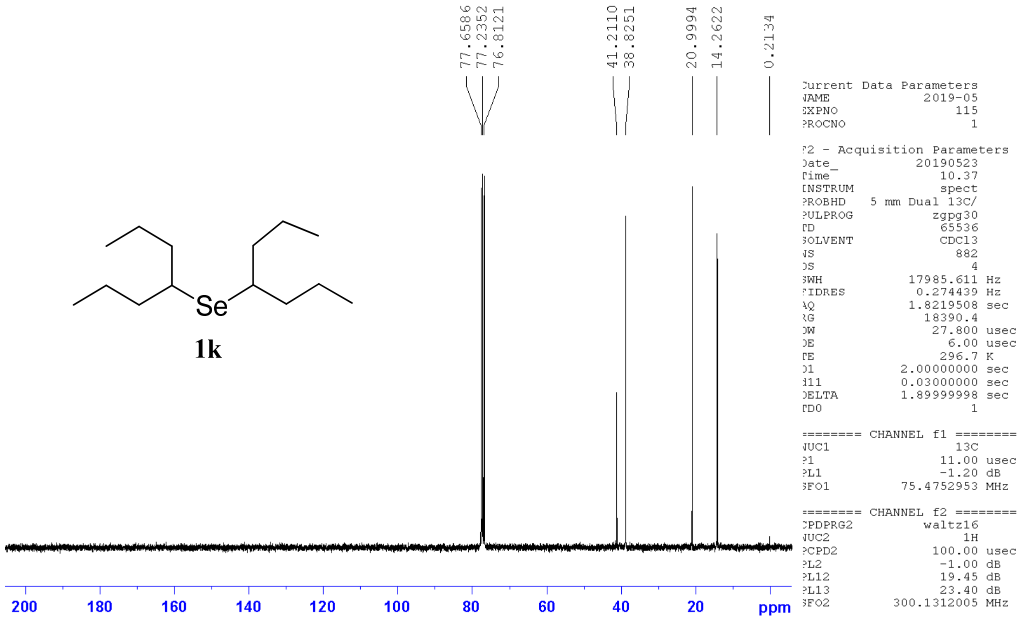

^{1}H NMR spectrum (300 MHz, $CDCl_3$) of compound 1k

^{13}C NMR spectrum (75 MHz, $CDCl_3$) of compound 1k

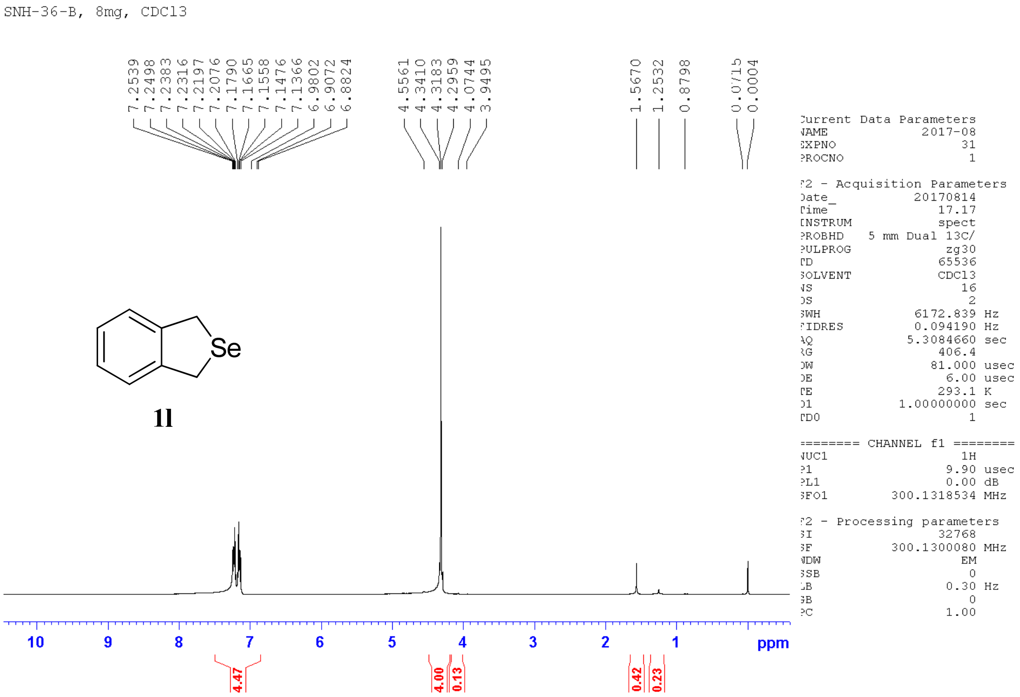

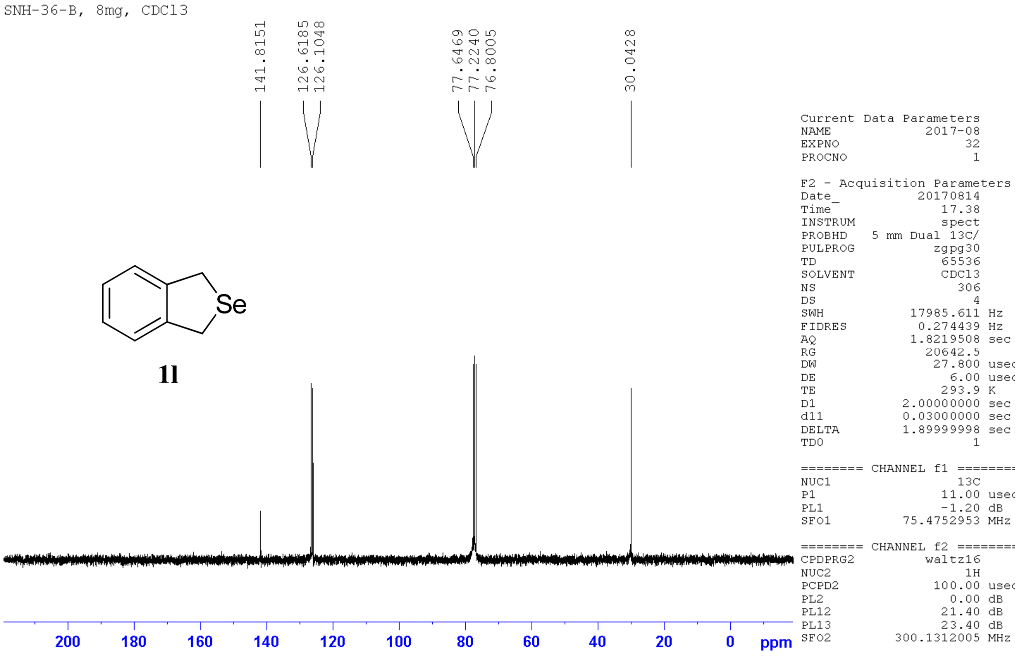

^{1}H NMR spectrum (300 MHz, $CDCl_3$) of compound 1l

^{13}C NMR spectrum (75 MHz, $CDCl_3$) of compound 1l

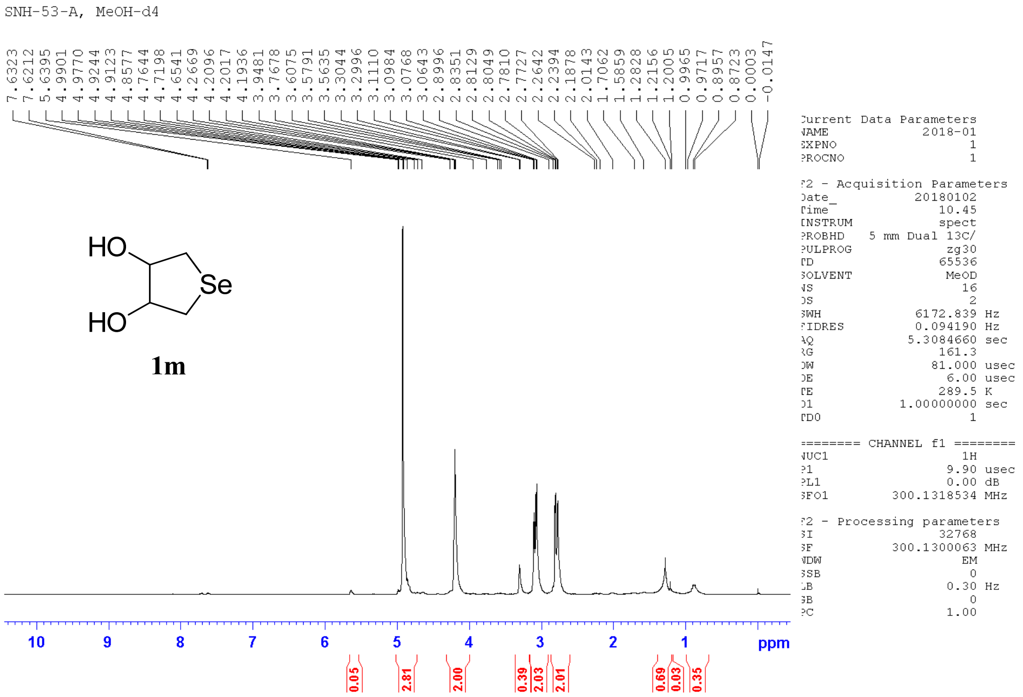

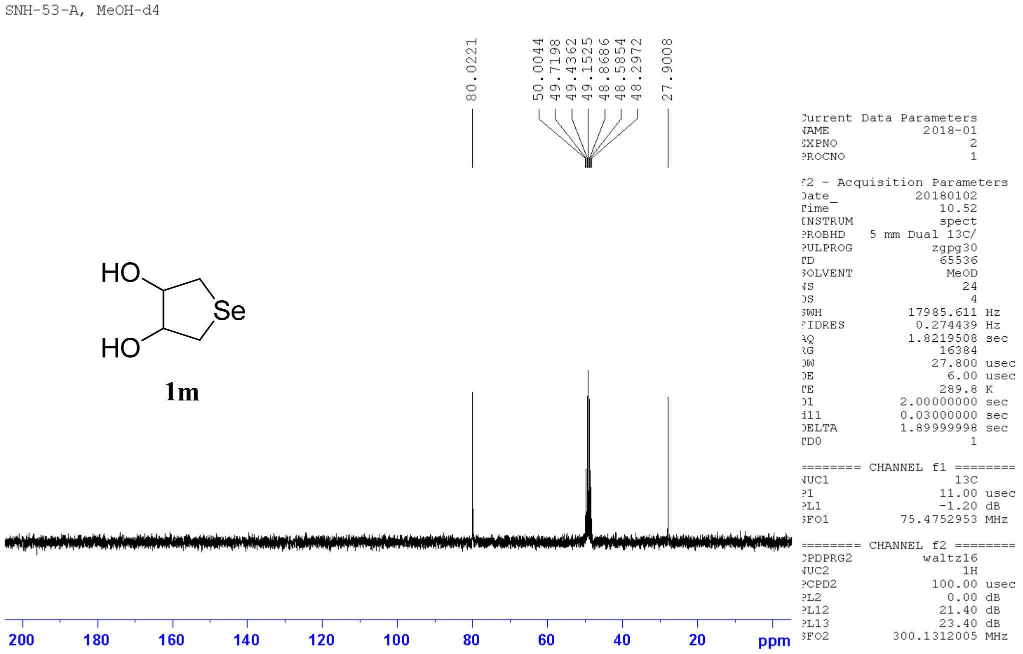

^{1}H NMR spectrum (300 MHz, MeOH-d4) of compound 1m

^{13}C NMR spectrum (75 MHz, MeOH-d4) of compound 1m
